# One year of laboratory-based COVID-19 surveillance system in Belgium: main indicators and performance of the laboratories (March 2020–21)

**DOI:** 10.1186/s13690-021-00704-2

**Published:** 2021-10-27

**Authors:** Marjan Meurisse, Adrien Lajot, Yves Dupont, Marie Lesenfants, Sofieke Klamer, Javiera Rebolledo, Tinne Lernout, Mathias Leroy, Arnaud Capron, Johan Van Bussel, Sophie Quoilin, Emmanuel Andre, Kaat Kehoe, Luc Waumans, Jos Van Acker, Olivier Vandenberg, Sigi Van den Wijngaert, Ann Verdonck, Lize Cuypers, Dieter Van Cauteren

**Affiliations:** 1 Scientific Directorate of Epidemiology and Public Health, Service Epidemiology of infectious diseases, Sciensano, J. Wytsmanstraat 14, 1050 Brussels, Belgium; 2 Quality of Laboratories, Sciensano, J. Wytsmanstraat 14, 1050 Brussels, Belgium; 3 Healthdata.be, Sciensano, J. Wytsmanstraat 14, 1050 Brussels, Belgium; 4 National Reference Center Respiratory Pathogens, Department of Laboratory Medicine, UZ Leuven, Herestraat 49, 3000 Leuven, Belgium; 5 Laboratory of Clinical Bacteriology and Mycology, Department of Microbiology, Immunology and Transplantation, Rega Institute, KU Leuven, 3000 Leuven, Belgium; 6 Department of Clinical and Molecular Pathology, AML, Sonic Healthcare, Antwerp, Belgium; 7 Clinical Laboratory, Jessa Hospital, Hasselt, Belgium; 8 Laboratory of Clinical Microbiology, AZ Sint-Lucas, Groenebriel 1, 9000 Ghent, Belgium; 9 Department of Microbiology, LHUB-ULB, Université Libre de Bruxelles, Brussels, Belgium; 10 Center for Environmental Health and Occupational Health, School of Public Health, Université Libre de Bruxelles (ULB), Brussels, Belgium; 11 Division of Infection and Immunity, Faculty of Medical Sciences, University College London, London, UK

**Keywords:** COVID-19, SARS-CoV-2, Belgium, Laboratory-based surveillance

## Abstract

**Background:**

With the spread of coronavirus disease 2019 (COVID-19), an existing national laboratory-based surveillance system was adapted to daily monitor the epidemiological situation of severe acute respiratory syndrome coronavirus 2 (SARS-CoV-2) in the Belgium by following the number of confirmed SARS-CoV-2 infections, the number of performed tests and the positivity ratio. We present these main indicators of the surveillance over a one-year period as well as the impact of the performance of the laboratories, regarding speed of processing the samples and reporting results, for surveillance.

**Methods:**

We describe the evolution of test capacity, testing strategy and the data collection methods during the first year of the epidemic in Belgium.

**Results:**

Between the 1^st^ of March 2020 and the 28^th^ of February 2021, 9,487,470 tests and 773,078 COVID-19 laboratory confirmed cases were reported. Two epidemic waves occurred, with a peak in April and October 2020. The capacity and performance of the laboratories improved continuously during 2020 resulting in a high level performance. Since the end of November 2020 90 to 95% of the test results are reported at the latest the day after sampling was performed.

**Conclusions:**

Thanks to the effort of all laboratories a performant exhaustive national laboratory-based surveillance system to monitor the epidemiological situation of SARS-CoV-2 was set up in Belgium in 2020. On top of expanding the number of laboratories performing diagnostics and significantly increasing the test capacity in Belgium, turnaround times between sampling and testing as well as reporting were optimized over the first year of this pandemic.

**Supplementary Information:**

The online version contains supplementary material available at 10.1186/s13690-021-00704-2.

## Background

The severe acute respiratory syndrome coronavirus 2 (SARS-CoV-2), identified in Wuhan end of December 2019, was first reported in Europe at the end of January 2020 [1–4]. The first coronavirus disease 2019 (COVID-19) case in Belgium was confirmed on the 3^rd^ of February 2020. This infected, but asymptomatic person, was one of the nine Belgians repatriated from Wuhan [5]. In March 2020, the first autochthonous infections were reported, followed by a sharp increase in the number of SARS-CoV-2 infections in Belgium [6].

With the spread of COVID-19, a close monitoring of this disease became necessary in order to inform public health decisions and actions. In the frame of its surveillance activities, the department of Epidemiology and public health of Sciensano, the Belgian institute for health, progressively implemented the daily monitoring of the epidemiological situation of SARS-CoV-2 in the country through multiple surveillance systems from February 2020 onwards. One of these surveillance systems is a national laboratory-based surveillance system [7, 8].

The main objective of the national laboratory-based surveillance system of COVID-19 is to daily monitor the epidemic in Belgium by describing trends of virus circulation in the population over time. The most important indicators produced by this surveillance system are the number of confirmed SARS-CoV-2 infections, the number of performed tests and the positivity ratio, by age and geographical occurrence.

In this paper, we present these indicators over a one-year period (1^st^ of March 2020 – 28^th^ of February 2021) in Belgium, taking into account the evolution of the laboratory test capacity and testing strategy during this period as well as the impact of performance of the laboratories regarding speed of processing the samples and reporting results to Sciensano for surveillance.

## Methods

### Test capacity

At the start of the COVID-19 epidemic early 2020, diagnostic testing for SARS-CoV-2 in Belgium was exclusively limited to the National Reference Centrum (NRC) for respiratory pathogens (University hospital of Leuven) [9]. With the rapid increase of the number of COVID-19 cases in the beginning of March, clinical microbiology laboratories (CML) also started to implement routine diagnosis of SARS-CoV-2 infection using in-house reverse transcription polymerase chain reaction (RT-PCR) assays. A certification process was implemented based on cross-validation of positive samples with the NRC and participation in an external quality assessment scheme organized under the supervision of Sciensano. Between the 1^st^ of March and the 1^st^ of May 2020, 73 laboratories across the country were certified for the molecular diagnosis of COVID-19 (68% of the laboratories currently certified). CML’s were also certified for the use of commercial antigenic tests as long as they used tests recognized by the federal agency for medicines and health products.

**Fig. 1:**
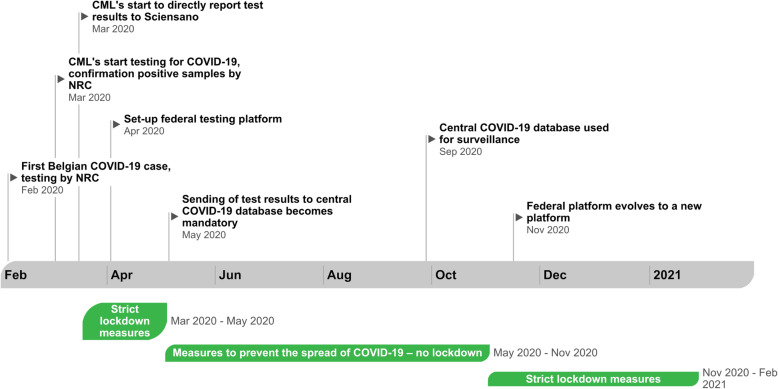
Overview of the laboratory-based COVID-19 surveillance outline and major social distancing measures (“lockdowns”) that were implemented, February 2020 – February 2021, Belgium

In reaction to the shortage of reagents and disposables, a federal testing platform was set-up in the beginning of April 2020 to further increase the test capacity in Belgium. This platform consisted of a consortium of university hospitals, biotech, and pharmaceutical companies across Belgium, and was initially primarily dedicated to perform testing in long-term care facilities, and later also in other residential collectivities and sampling-triage centers [10]. In addition to increasing the test capacity, this platform also allowed to partially compensate the overflow encountered at CML level. It evolved in November 2020 to a new platform composed of 8 university laboratories linked to CML’s qualified to perform SARS-CoV-2 RT-PCR assays (Fig. 1). These laboratories are geographically spread across the country to minimize logistic delays.

### Data collection and reporting

Initially all positive samples for SARS-CoV-2 were sent to the NRC UZ Leuven for confirmation and the latter reported the confirmed COVID-19 cases to Sciensano. From the 15^th^ of March 2020 onwards, laboratories implemented daily reporting of test results directly to Sciensano without confirmation by the NRC [11]. Since April 2020, test results of the federal testing platform were added to the test results reported by the NRC and the recognized clinical laboratories for molecular diagnosis of COVID-19 [12] (Fig. 1).

The implementation of a new laboratory-based surveillance system and data collection was built upon the experience of the Belgian Sentinel Network of Laboratories (BSNL), established in 1983. This network, coordinated by Sciensano, is used to collect data on a weekly basis from laboratory confirmed cases of about 30 infectious diseases, reported by the participating sentinel laboratories [7, 13, 14]. For the laboratory-based surveillance of COVID-19, daily reporting of all test results performed by CML’s became mandatory for reimbursement [15–17] instead of the weekly and voluntary reporting of positive cases usually used by the BSNL [7].

Laboratories were required to report positive, negative and inconclusive test results of RT-PCR and antigenic diagnostic tests. Data on a selection of variables was collected as for the BSNL network: sampling date, date of test result, test result, type of diagnostic test and patient demographic variables that allow identification of duplicate cases (i.e. postal code, date of birth and gender). Every day, from the beginning of March 2020 until the end of September 2020 incoming data from all recognized laboratories for COVID-19 testing in Belgium through the BSNL dataflow were processed by researchers at Sciensano and all data were centralized, stored on an SQL server and used for reporting.

Since the 5^th^ of May 2020, laboratories (NRC, CML’s and the federal testing platform) were additionally asked to report their COVID-19 test results to the healthdata.be platform of Sciensano, in response to the launch of the national contact-tracing system [15]. From the end of September onwards, this laboratory data collection was also used for surveillance and replaced the COVID-19 data collection via the BSNL network. Laboratories had to record test results in their Laboratory Information System and complete the mandatory fields of the COVID-19 Laboratory-Test-Result form according to defined technical specifications [18]. The collected variables were similar to those reported for the BSNL network but included as well nominal data necessary for contact-tracing. Laboratories were required to report positive, negative and inconclusive test results of RT-PCR, antigenic and serological diagnostic tests, ideally within 1 to 4 hours after validation of the test in the laboratory [15]. The test results were collected and stored in a central COVID-19 database of the healthdata.be platform.

A data cleaning process was executed daily. Persons could be tested more than once and therefore this process included a de-duplication step for COVID-19 laboratory confirmed cases. Initially, only the first positive test result since the beginning of the epidemic was considered as a new COVID-19 laboratory confirmed case. At the end of October 2020, the deduplication process was adapted based on the advice the Risk Assessment Group (RAG) [19] and the first positive test result within an eight-week period was taken into account as a new COVID-19 laboratory confirmed case. After the data cleaning process, a subset of the test results and de-duplicated COVID-19 laboratory confirmed cases of the last seven reported days was created. These subsets contained only an anonymous selection of variables that was pushed daily through a secured data transfer procedure and merged with the historical datasets of test results and COVID-19 laboratory confirmed cases.

Data on COVID-19 laboratory confirmed cases and performed COVID-19 diagnostic tests have been reported to the public and authorities on a daily and weekly basis by Sciensano in different epidemiological bulletins since 14 March 2020 [20], as well as via open data and on an interactive dashboard [21]. Key indicators and trends are shown (e.g. the average of new laboratory confirmed cases for Belgium of the last 7 days vs. the 7 days before, the number of tests performed per day and the evolution of the positivity ratio by age group and geographical distribution at national, regional and provincial level).

### Testing strategy

**Fig. 2:**
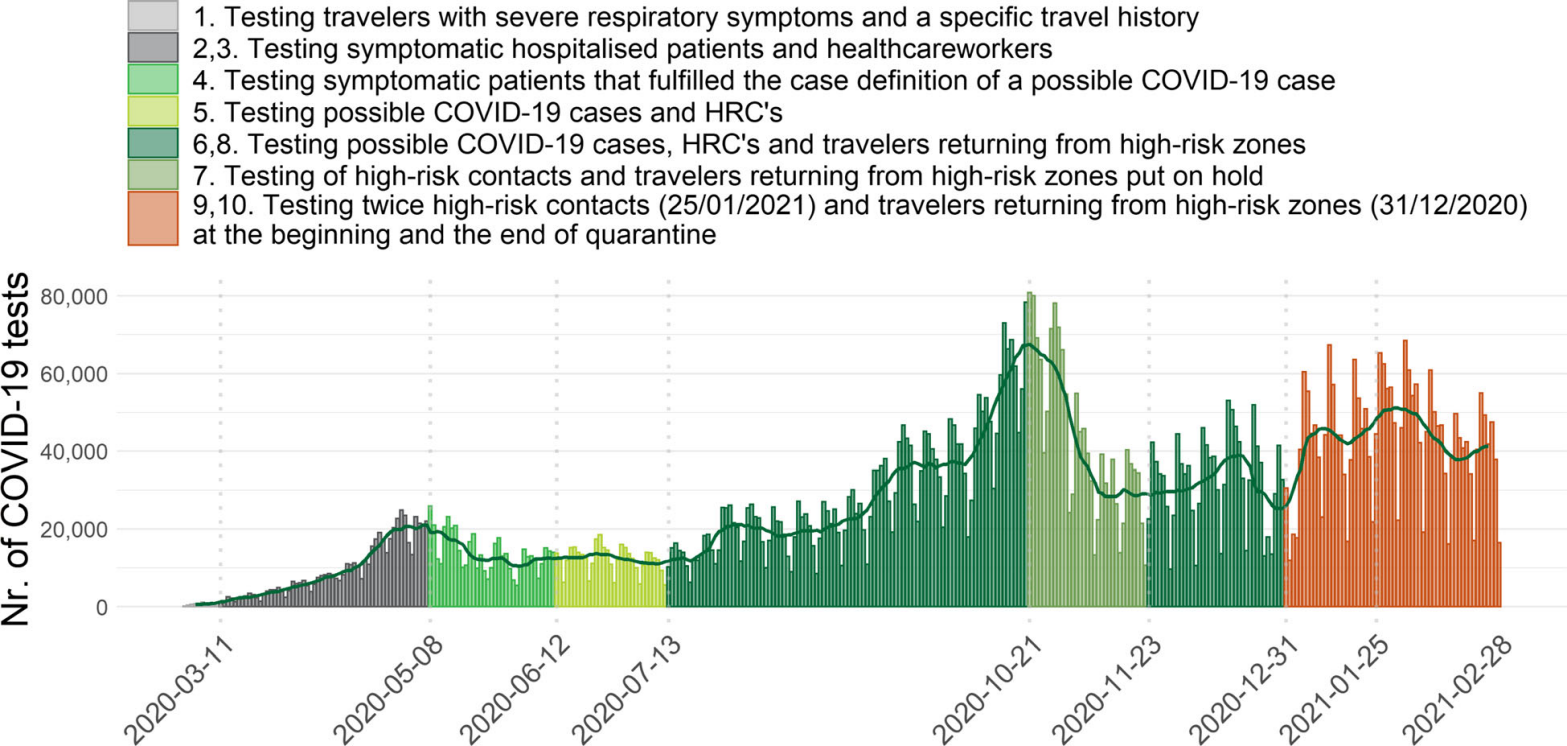
Number of COVID-19 diagnostic tests reported per day and the moving average over seven centered days (dark green line) per date of test result, from the 1^st^ of March 2020 until the 28^th^ of February 2021, Belgium. Bars are colored regarding the implemented testing strategy and the legend is numbered in correspondence to the numbered testing strategies in Table 1

Testing criteria for RT-PCR testing have evolved during the course of the epidemic in Belgium, taking into account the epidemiological situation and the available test capacity. The main testing strategies are presented in Table 1 and Fig. 2. They were implemented at national level and identical for all Belgian regions.

**Table 1:** Overview of the main testing strategies in Belgium since the beginning of the epidemic

Timing	Strategy
Beginning epidemic	1. Testing travelers with severe respiratory symptoms returning from areas with recognized local transmission during the last 14 days or symptomatic patients that has physical contact with a laboratory confirmed COVID-19 case.
11 March 2020	2. Travel history dropped as criteria for testing. Exclusively testing of hospitalized cases with acute respiratory symptoms and healthcare workers with acute respiratory symptoms and fever [22].
28 March 2020	3. Recommendation to test the first cases (maximum 5) of a cluster in a residential collectivity fitting the case definition of a possible COVID-19 case.
8 May 2020	4. Testing of all symptomatic patients that fulfilled the case definition of a possible COVID-19 case [23].
12 June 2020	5. Testing of all symptomatic patients that fulfilled the case definition of a possible COVID-19 case and high risk contacts (HRC) of a COVID-19 case (one test at the moment of identification, and a second test at the end of quarantine for health care workers) [22].
13 July 2020	6. Testing strategy broadened to include travelers arriving in Belgium from areas abroad considered as high risk zones (single test).
21 October 2020	7. Testing of asymptomatic HRC and travelers returning from high risk areas abroad temporarily put on hold, because of insufficient test capacity.
23 November 2020	8. Restart testing of asymptomatic HRC and travelers returning from high risk areas abroad.
31 December 2020	9. Two tests need to be performed for travelers returning from high risk areas abroad as soon as possible after arrival and at day 7 after the day of return.
25 January 2021	10. Two tests need to be performed for HRC at the moment of identification and at day 7 after the day of last high risk contact.

## Results

### Number of tests

Between the 1^st^ of March 2020 and the 28^th^ of February 2021, 9,487,470 tests (RT-PCR or antigen) were reported through the national laboratory-based surveillance system. Figure 2 shows the number of reported tests per day during this period. The highest number of tests were performed towards the end of October 2020, reaching 80,000 tests a day. These results represents almost only RT-PCR tests as antigen tests were not widely used in Belgium during this period (98.6% RT-PCR, 1.4% antigen tests).

### Number of laboratory confirmed cases and positivity ratio

**Fig. 3:**
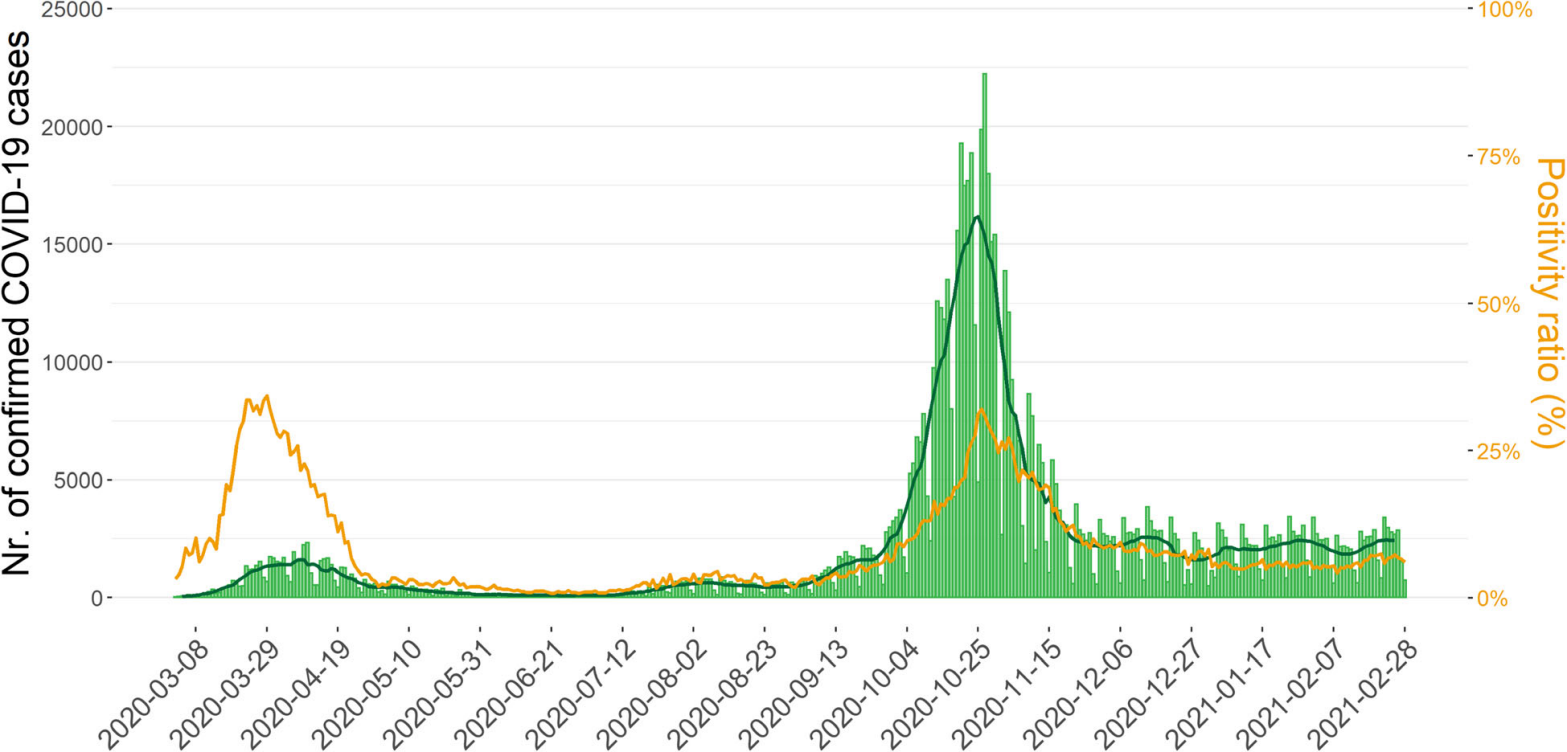
Daily number of COVID-19 laboratory confirmed cases (light green bars), the moving average over seven days (dark green line) and positivity ratio (orange line), from the 1^st^ of March 2020 until the 28^th^ of February 2021, Belgium

Between the 1^st^ of March 2020 and the 28^th^ of February 2021 a total of 773,078 COVID-19 laboratory confirmed cases were reported. During this period two epidemic waves occurred, with the peak of the first and second wave on the 10^th^ of April with 2336 cases and the 27^th^ of October 2020 with 22,214 cases respectively (Fig. 3).

### Turnaround time (TAT) between sampling and test result

**Fig. 4:**
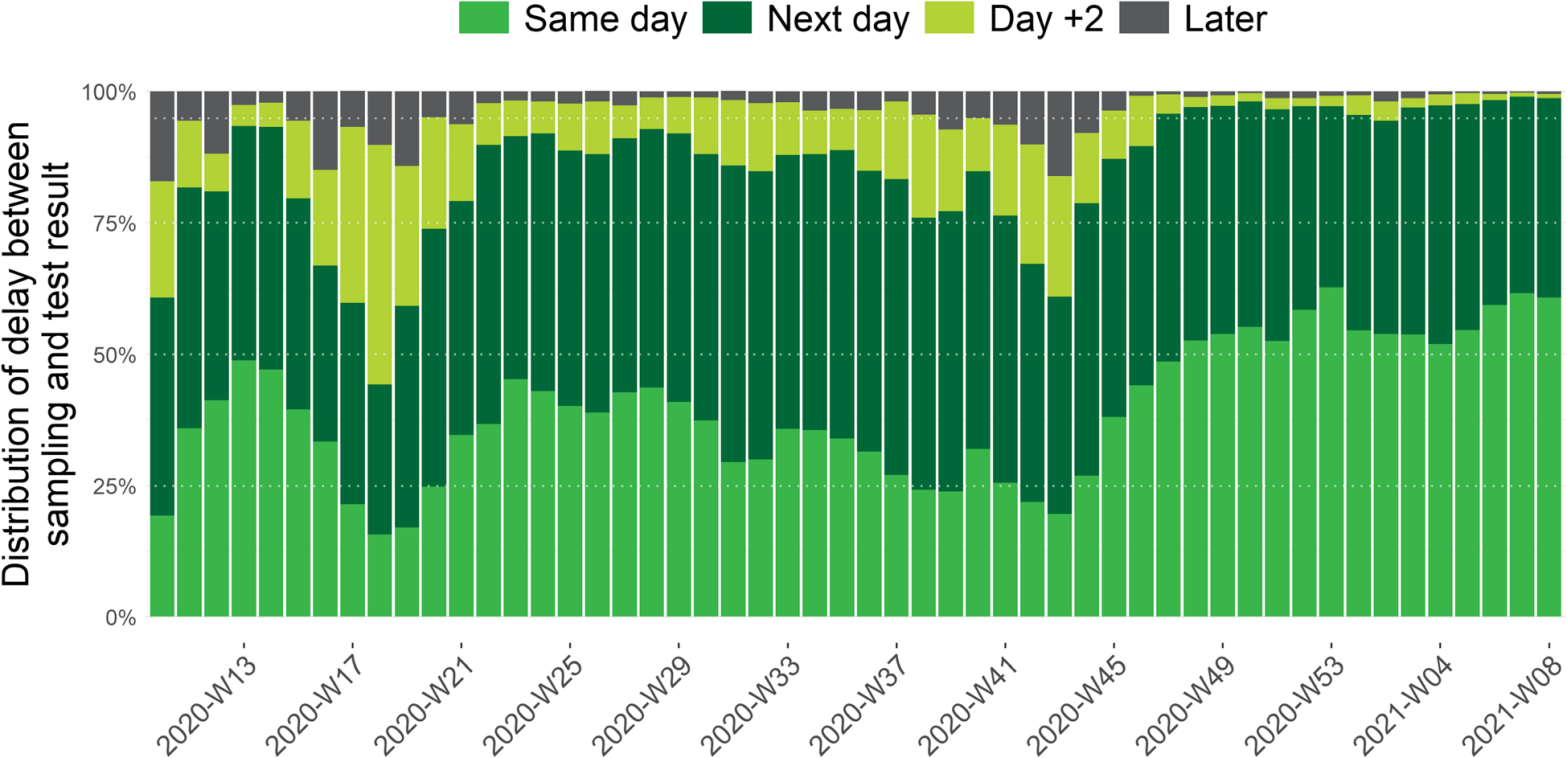
Weekly proportion of COVID-19 tests by delay between date of sampling and date of test result, from the 1^st^ of March 2020 (week 10) until the 28^th^ of February 2021 (week 08), Belgium

Figure 4 shows the evolution of the weekly percentage of tests with a test result within different timeframes after the date of sampling. Since mid-November 2020 (week 48) until the end of February 2021, a test result is available at the latest the day after sample collection for 95% of the tests performed and for more than 50% of the tests the same day.

### Turnaround time between test result and reporting

**Fig. 5:**
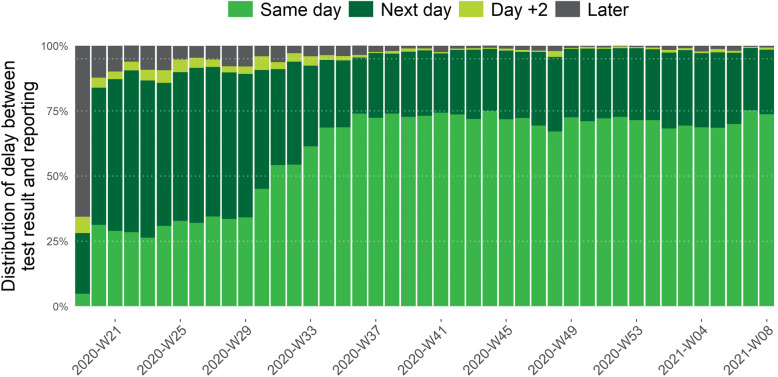
Weekly proportion of COVID-19 tests by delay between date of test result and date of reporting, from the 5^th^ of May 2020 (week 19) until the 28^th^ of February 2021 (week 08), Belgium

Figure 5 shows the TAT between the availability of the test result and its reporting to the healthdata.be platform of Sciensano since the implementation of the central COVID-19 database for contact tracing in the beginning of May 2020 (week 19). We see an increase of the speed of reporting that remains stable since the end of August 2020 with almost 75% of test results that are reported within the same day and more than 95% that are reported at the latest the day after obtaining the test result.

### Turnaround time between sampling and reporting

**Fig. 6:**
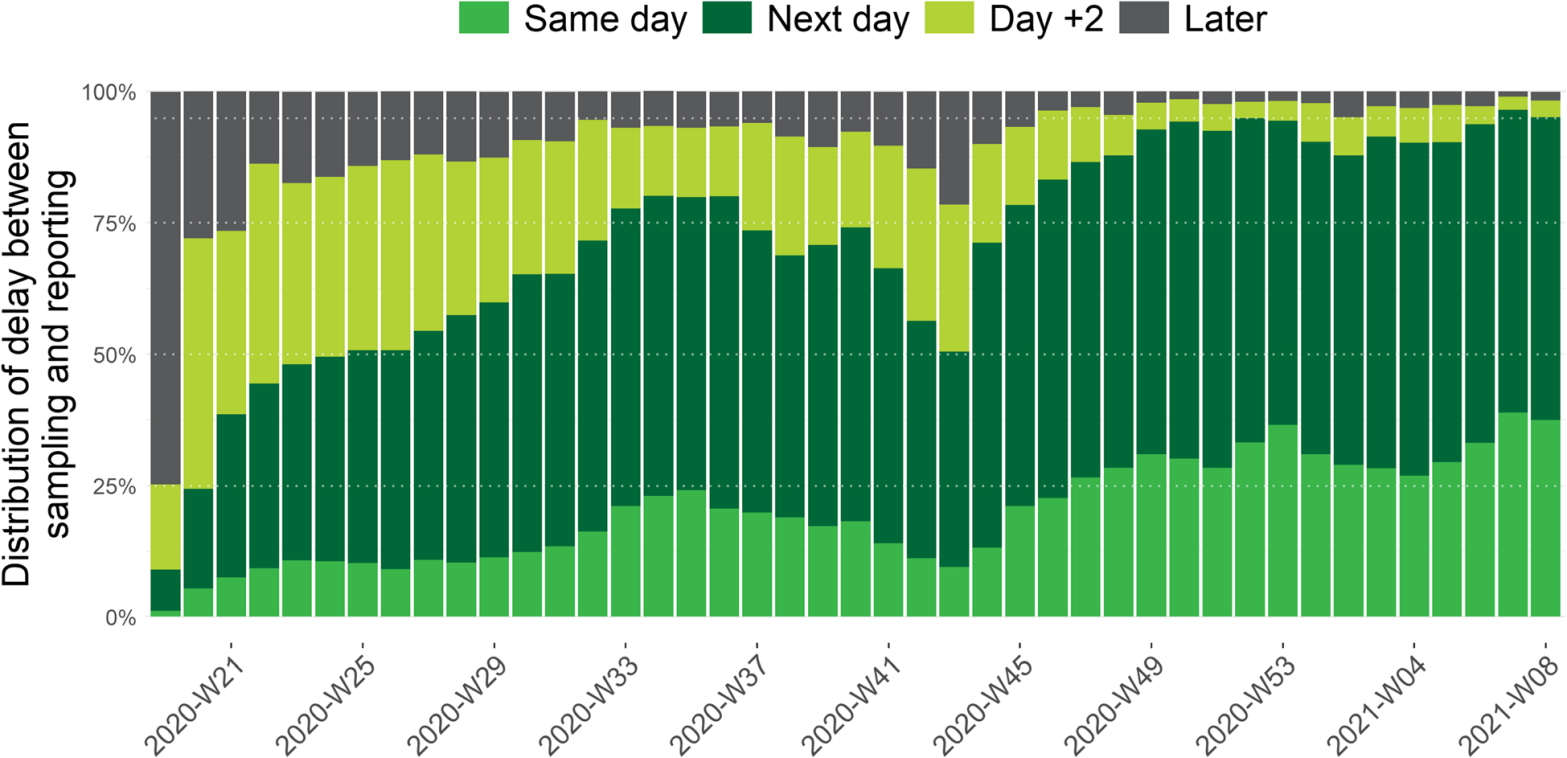
Weekly proportion of COVID-19 tests by delay between date of sampling and date of reporting, from the 5^th^ of May 2020 (week 19) until the 28^th^ of February 2021 (week 08), Belgium

Combining both TAT’s above, Fig. 6 shows the overall TAT between sampling and the reporting of the test result to the healthdata.be platform of Sciensano since the implementation of the central COVID-19 database for contact tracing (week 19). We see a decrease of this TAT during the summer period and an increase during the second wave at the end of October that improved again from week 44, with 90 to 95% of test results that are reported at the latest the day after sampling since week 49.

## Discussion

In the context of the SARS-CoV-2 pandemic, the laboratory-based surveillance of COVID-19, together with the national hospital-based surveillance [24] and mortality surveillance [22], was crucial to follow the evolution of the SARS-CoV-2 epidemic in Belgium. Monitoring the number of reported COVID-19 cases and performed tests in Belgium allowed to follow-up key indicators and trends, and allowed to provide this information to the different authorities for policy making.

The participation of CML’s in Belgium has been essential since the beginning of the epidemic. An important number of CML’s throughout the country were certified between March and May 2020 and as a result, the number of tests performed increased significantly. Because the availability of large-scale testing is crucial for monitoring the progression of the epidemic, Belgium decided to also rely on a federal platform to increase testing capacities. This combined testing strategy is halfway between the German strategy relying on a clinical laboratory network and the centralized testing approach developed by the UK government [25, 26]. Thanks to both the high number of laboratories performing testing and the accompanied increasing laboratory test capacity, the testing strategy could be broadened at different stages during spring 2020. Overall, almost 10 million tests have been performed by the NRC, CML’s and federal platforms over 1 year (Belgium has 11.5 million inhabitants). As CML’s need to be certified to perform diagnostic testing and report all results in a timely matter in order to be reimbursed, we believe this number reflects the true number of diagnosed cases in Belgium. However testing criteria have evolved during the course of the epidemic with a direct impact on the ability to detect (a)symptomatic cases, e.g. testing of all HRC is performed since June 2020 (with an interruption between the 21^st^ of October and the 23^rd^ of November 2020).

Results of antigenic tests represented only 1.4% of the tests performed in Belgium during the study period. Between April and August 2020, the use of these tests was generally not recommended, with limited use to situations where no RT-PCR could be performed, or as a first diagnostic/screening test to isolate positive individuals if an RT-PCR could only be performed at a later time point. RT-PCR should anyway be performed as soon as possible on all samples with negative antigen test [27]. Since November 2020, rapid antigenic tests performed by a healthcare professional can be used in specific situations e.g. symptomatic patients with recent onset of illness (≤ 5 days), investigation of clusters (testing of low-risk contacts) and repetitive screening of asymptomatic persons [28].

The efforts done by the laboratories to increase test capacity, as well as logistic improvements, also resulted in an improvement of the TAT between date of sampling and laboratory test result and the speed of reporting of these results to Sciensano to be used for surveillance and to initiate contact tracing. After the first wave, from the end of April to end of May 2020, there was an improvement of the TAT between sampling and result that was followed by an improvement of the speed of reporting to Sciensano in July–August 2020. As a result, from May to the end of August 2020 the TAT between sampling and reporting improved continuously to reach more than 75% of the test results that were reported at the latest the day after the day of sampling. The second wave, with a peak in October 2020, resulted in a high number of performed tests, impacting the sampling facilities and laboratories and resulting in an increase of the TAT between sampling and test result. In this context, it was decided to prioritize and temporary stop testing of asymptomatic high-risk contacts from week 44 to week 47. This resulted in a clear improvement again of the TAT. Following the control measures taken, the number of cases and the number of tests progressively declined starting from week 45 of 2020.

Since the end of November 2020, 90 to 95% of test results are reported at the latest the day after sampling. This high performance continued in January–February 2021, although there was an increase in the number of tests carried out, indicating the ability of the testing facilities and laboratories to quickly process and report a high number of tests.

Despite these achievements over the past year, new challenges are arising for 2021. The data collection of laboratory test results has been modified in March 2021 to allow reporting of semi-quantitative interpretation of cycle threshold (Ct) values to gain knowledge on the dynamics of the epidemic [29]. Rapid diagnostic tools such as antigenic tests will be more broadly used for testing outside laboratories settings. It is essential that positive results of these tests are reported as systematically and as quickly as the laboratory reporting. These tests however also present limitations [28, 30] and the involvement of the clinical laboratories will remain crucial for confirmation of test results and monitoring the laboratory test capacity and the positivity rate in 2021.

The real time integration of genomic data provided by CML’s and/or large sequencing platforms is another challenge that will be crucial to better track community spread and transmission chains. Genomic data will help to identify viral mutations, and combined with health data could inform about viral genome correlations with clinical outcomes [31]. Reporting of genomic data from testing facilities to Sciensano is possible since March 2021 to take up this challenge [32, 33].

Finally, with the start of the vaccination campaign in Belgium in 2021, laboratories will be essential for the detection and monitoring of breakthrough cases post-vaccination. We can build on the experience and all the work done during the first year of the epidemic to continue to evolve and improve our monitoring through a laboratory-based COVID-19 surveillance system, towards a data warehouse centralizing and linking testing (inside and outside laboratories), contact tracing, hospitalization and vaccination data.

## Conclusion

Thanks to the effort of all laboratories and built upon an existing laboratory network, a performant exhaustive national laboratory-based surveillance system to monitor COVID-19 was set up in Belgium in 2020. On top of expanding the number of laboratories performing diagnostics and significantly increasing the test capacity in Belgium, turnaround times between sampling and testing as well as reporting were optimized continuously over the first year of this pandemic.

## Supplementary Information

**Additional file 1.** Turnaround time between sampling and test result. Weekly number of tests with a test result within different timeframes after the date of sampling.

**Additional file 2.** Turnaround time between test result and reporting. Weekly number of tests with the reporting of the test result to the healthdata.be platform of Sciensano within different timeframes after the date of the test result.

**Additional file 3.** Turnaround time between sampling and reporting. Weekly number of tests with the reporting of the test result to the healthdata.be platform of Sciensano within different timeframes after the date of sampling.

## Data Availability

The datasets supporting the conclusions of this article are available in the Epistat repository, https://epistat.wiv-isp.be/covid/ or included within the article’s additional files.

## Abbreviations

COVID-19: Coronavirus disease 2019
SARS-CoV-2: Severe acute respiratory syndrome coronavirus 2
NRC: National Reference Centrum
CML: Clinical microbiology laboratory
RT-PCR: Reverse transcription polymerase chain reaction
BSNL: Belgian Sentinel Network of Laboratories
TAT: Turnaround time
Ct: Cycle threshold

## References

[1] World Health Organization (2020). Novel Coronavirus (2019-nCoV) situation report - 1 21 January 2020.

[2] Singhal T (2020). A review of coronavirus Disease-2019 (COVID-19). Indian J Pediatr.

[3] World Health Organization (2020). Novel Coronavirus (2019-nCoV) situation report - 6 26 January 2020.

[4] Spiteri G, Fielding J, Diercke M, Campese C, Enough V, Gaymard A (2020). First cases of coronavirus disease 2019 (COVID-19) in the WHO European region, 24 January to 21 February 2020. Euro Surveill.

[5] Federal Public Service (FPS) Health, Food Chain Safety and Environment (2020). One repatriated Belgian has tested positive for the novel coronavirus.

[6] Sciensano. Covid-19 - situation epidemiologique au 14 mars 2020: Sciensano; 2020. Available from: https://covid-19.sciensano.be/sites/default/files/Covid19/COVID-19_Daily%20report_20200314%20-%20FR.pdf.

[7] Muyldermans G, Ducoffre G, Leroy M, Dupont Y, Quolin S (2016). Participating sentinel laboratories T, et al. surveillance of infectious diseases by the sentinel laboratory network in Belgium: 30 years of continuous improvement. PLoS One.

[8] Walckiers D, Stroobant A, Yourassowsky E, Lion J, Cornelis R (1991). A sentinel network of microbiological laboratories as a tool for surveillance of infectious diseases in Belgium. Epidemiol Infect.

[9] Weemaes M, Martens S, Cuypers L, Van Elslande J, Hoet K, Welkenhuysen J (2020). Laboratory information system requirements to manage the COVID-19 pandemic: a report from the Belgian national reference testing center. J Am Med Inform Assoc.

[10] Hoxha A, Wyndham-Thomas C, Klamer S, Dubourg D, Vermeulen M, Hammami N, et al. Asymptomatic SARS-CoV-2 infection in Belgian long-term care facilities. Lancet Infect Dis. 2020:21, e67. 10.1016/S1473-3099(20)30560-0 PMC7333983 32628906

[11] Sciensano (2021). COVID-19 surveillance frequently asked questions.

[12] Capron A (2020). Lijst van laboratoria met diagnostiek voor covid-19.

[13] Berger N, Muyldermans G, Dupont Y, Quoilin S (2016). Assessing the sensitivity and representativeness of the Belgian sentinel network of laboratories using test reimbursement data. Arch Public Health.

[14] Sciensano (2020). Peillaboratoria.

[15] Van Oyen H, De Cock J (2020). OMZENDBRIEF AAN LABORATORIA 20 juli 2020 - De verplichte rapportage van PCR/antigeen en serologische resultaten in het kader van de COVID-19-pandemie.

[16] Sciensano (2020). Erkenningsprocedure van de medische laboratoria erkend door de FOD volksgezondheid voor het uitvoeren van de COVID-19 screeningstest met moleculair biologische technieken.

[17] RIZIV (2020). Omzendbrief laboratoria - Terugbetaling opsporingstesten Coronavirus tijdens de Covid-19 pandemie.

[18] healthdata.be (2020). Database COVID-19 test results - Technical guidelines.

[19] RAG (2020). Reinfection - update October 2020.

[20] Sciensano (2021). COVID-19 - Epidemiologische situatie.

[21] Sciensano (2021). Belgium COVID-19 Epidemiological Situation.

[22] Bustos Sierra N, Bossuyt N, Braeye T, Leroy M, Moyersoen I, Peeters I (2020). All-cause mortality supports the COVID-19 mortality in Belgium and comparison with major fatal events of the last century. Arch Public Health.

[23] Regeringscommissariaat Corona (2020). Note: stratégies de testing / Testing strategie.

[24] Van Goethem N, Vilain A, Wyndham-Thomas C, Deblonde J, Bossuyt N, Lernout T (2020). Rapid establishment of a national surveillance of COVID-19 hospitalizations in Belgium. Arch Public Health.

[25] Banatvala J (2020). COVID-19 testing delays and pathology services in the UK. Lancet.

[26] Kluge S, Janssens U, Welte T, Weber-Carstens S, Marx G, Karagiannidis C (2020). German recommendations for critically ill patients with COVID-19. Med Klin Intensivmed Notfallmedizin.

[27] RAG (2020). Testing strategy update August 2020: pooling, saliva testing, RT-lamp, rapid antigen testing, self-collected nose, throat and nasopharyngeal swabs and multiplex.

[28] Sciensano (2021). Tests rapides antigéniques (Ag).

[29] Sciensano (2020). RAG interpretation and reporting of SARSCOV-2 PCR results.

[30] Schildgen V, Demuth S, Lüsebrink J, Schildgen O. Limits and opportunities of SARS-CoV-2 antigen rapid tests – an experience based perspective: medRxiv. Cold Spring Harbor Laboratory Press; 2020. 2020.09.22.20199372 10.3390/pathogens10010038 PMC7824818 33466537

[31] Vandenberg O, Martiny D, Rochas O, van Belkum A, Kozlakidis Z (2021). Considerations for diagnostic COVID-19 tests. Nat Rev Microbiol.

[32] healthdata.be (Sciensano) (2021). Database covid-19 test results.

[33] healthdata.be (Sciensano) (2021). Laboratory test result variants.

